# Numerical Simulation of NH_3_(CH_2_)_2_NH_3_MnCl_4_ Based Pb-Free Perovskite Solar Cells Via SCAPS-1D

**DOI:** 10.3390/nano12193407

**Published:** 2022-09-28

**Authors:** Khursheed Ahmad, Waseem Raza, Rais Ahmad Khan, Ali Alsalme, Haekyoung Kim

**Affiliations:** 1 School of Materials Science and Engineering, Yeungnam University, Gyeongsan 38541, Korea; 2 Department of Chemical Engineering, Indian Institute of Technology Delhi, Hauz Khas, New Delhi 110016, India; 3 Department of Chemistry, College of Science, King Saud University, Riyadh 11451, Saudi Arabia

**Keywords:** NH_3_(CH_2_)_2_NH_3_MnCl_4_, Pb-free perovskite solar cells, electron transport layer, numerical simulation, SCAPS-1D

## Abstract

Recently, the design and fabrication of lead (Pb)-free perovskite or perovskite-like materials have received great interest for the development of perovskite solar cells (PSCs). Manganese (Mn) is a less toxic element, which may be an alternative to Pb. In this work, we explored the role of NH_3_(CH_2_)_2_NH_3_MnCl_4_ perovskite as a light absorber layer via SCAPS-1D. A Pb-free PSC device (FTO/TiO_2_/NH_3_(CH_2_)_2_NH_3_MnCl_4_/spiro-OMeTAD/Au) was simulated via SCAPS-1D software. The simulated Pb-free PSCs (FTO/TiO_2_/NH_3_(CH_2_)_2_NH_3_MnCl_4_/spiro-OMeTAD/Au) showed decent power conversion efficiency (PCE) of 20.19%. Further, the impact of the thickness of absorber (NH_3_(CH_2_)_2_NH_3_MnCl_4_), electron transport (TiO_2_), and hole-transport (spiro-OMeTAD) layers were also investigated. Subsequently, various electron transport layers (ETLs) were also introduced to investigate the role of ETL. In further studies, an NH_3_(CH_2_)_2_NH_3_MnCl_4_-based PSC device (FTO/TiO_2_/NH_3_(CH_2_)_2_NH_3_MnCl_4_/spiro-OMeTAD/Au) was also developed (humidity = ~30–40%). The fabricated PSCs displayed an open circuit voltage (Voc) of 510 mV with a PCE of 0.12%.

## 1. Introduction

Perovskite solar cells (PSCs) are the most efficient next-generation photovoltaic devices and have received tremendous attention [1,2,3,4]. PSCs are comprised of different components in which perovskite perovskite-like materials are used as light absorbers [5,6,7,8,9]. In general, organic–inorganic hybrid perovskite materials can be represented with the molecular formula of ABX_3_ (where A = MA^+^, FA^+^, NH_4_^+^, Cs^+^; M = Pb+, Sn^2+^, or Ge^2+^; and X = Cl, Br^−^, I^−^) [10,11,12,13,14,15,16,17,18]. In 2009, Kojima et al. [5] introduced methyl ammonium lead halide (MAPbX_3_) perovskite as a visible light sensitizer for the development of dye-sensitized solar cells and obtained a power conversion efficiency (PCE) of less than 4%. Further, enormous efforts/strategies were made by the scientific community to enhance the performance of the MAPbX_3_-based PSCs [19]. Recently, Zhang et al. [20] employed novel approaches and strategies and reported an excellent PCE of 20.1%. In another report, the highest PCE of more than 25% has been published [4]. This excellent PCE makes PSCs a potential candidate for practical applications, but the presence of toxic Pb and poor aerobic stability of MAPbX_3_ restricts its large-scale production [3]. Thus, it is of great importance to design and fabricate Pb-free PSCs. Methyl ammonium tin iodide (MASnI_3_) has a narrow band gap and excellent absorption coefficient, which suggests its potential application as a Pb-free light absorber for the development of PSCs [16]. In 2014, Snaith and co-workers reported a promising PCE of more than 6% using MASnI_3_ as a light absorber [16]. Further, various strategies and efforts were made to enhance the PCE of MASnX_3_-based PSCs, and the highest PCE of more than 11% was achieved, but instability of the Sn^2+^ ion in the MASnI_3_ structure is still a major concern [17]. In this regard, researchers have investigated the optoelectronic properties of other Pb-free materials such as MA_3_Bi_2_I_9_, Cs_3_Bi_2_I_9_, MA_3_Sb_2_I_9_, C_6_H_4_NH_2_CuBr_2_I, Cs_3_Sb_2_I_9_, and (NH_4_)_3_Sb_2_I_9_ [15,21,22,23,24,25,26]. The developed PSCs with such perovskite-like materials exhibited poor PCE, which may be due to the wide band gap of these perovskite-like materials. Hence, it is desirable to design or find out other Pb-free low band gap perovskite or perovskite-like materials. Manganese (Mn)-based perovskite material (NH_3_(CH_2_)_2_NH_3_MnCl_4_) has a band gap of ~1.81 eV, and it can be employed as a light absorber material for the development of Pb-free PSCs [27].

Herein, we have investigated the role of NH_3_(CH_2_)_2_NH_3_MnCl_4_ as a suitable light absorber for the development of Pb-free PSCs via SCAPS-1D. We have designed and simulated Pb-free PSCs (FTO/ETL/NH_3_(CH_2_)_2_NH_3_MnCl_4_/spiro-OMeTAD/Au via a one-dimensional Solar Cell Capacitance-Simulator (SCAPS-1D). The effect of various thicknesses of (ETL), hole transport layer (HTL), and light absorber layers were studied to obtain the highest PCE. The simulated PSC device exhibited decent photovoltaic performance via SCAPS-1D. Additionally, we developed PSCs (FTO/c-TiO_2_/m-TiO_2_/NH_3_(CH_2_)_2_NH_3_MnCl_4_/Spiro-OMeTAD/Au), and their photovoltaic performance was investigated. 

## 2. Experimental

### 2.1. Device Simulation and Investigations

The SCAPS-1D developed by Prof. M. Burgelman [28] was used to simulate the PSCs. The SCAPS-1D software is generally based on the principle of Poisson’s/continuity equations, which are listed below:

∇^2^ψ = q/ε (n − p + N_A_ − N_D_) (1)

where N_D_ is the donor concentration, N_A_ is the acceptor concentration, and ψ is electrostatic-potential. 

The equations of continuity can be explained as given below,

∇·J_n_ − q ∂n/∂t = + qR (2)

where J_n_ is the current density of electrons and R is the rate of carrier recombination.

∇·J_P_ + q ∂p/∂t = −qR (3)

where J_p_ is the current density of holes.

Moreover, the Drift–Diffusion Current Relations can be described by Equations (4) and (5) as given below,

J_n_ = qnµ_n_E + qD_n_ ∇n (4)

where D_n_ is the diffusion coefficient of an electron.

J_p_= qpµ_p_E − qD_p_ ∇p (5)

where D_p_ is the hole diffusion coefficient.

The numerical simulation of NH_3_(CH_2_)_2_NH_3_MnCl_4_ based Pb-free PSCs was performed on SCAPS-1D (1 sun conditions, AM 1.5 G, 100 mW/cm^2^, and temperature 300 K). The input parameters (band gap, dielectric permittivity, electron affinity, electron/hole mobility, electron/hole thermal velocity, defect density, etc.) for FTO, NH_3_(CH_2_)_2_NH_3_MnCl_4_, TiO_2_, ZnO, ZnSe, WO_3_, WS_2_, SnO_2_, spiro-OMeTAD were taken from the previously reported literature [21,27,29,30,31,32] and have been presented in Tables S1 and S2.

### 2.2. Device Structure

**Scheme 1:**
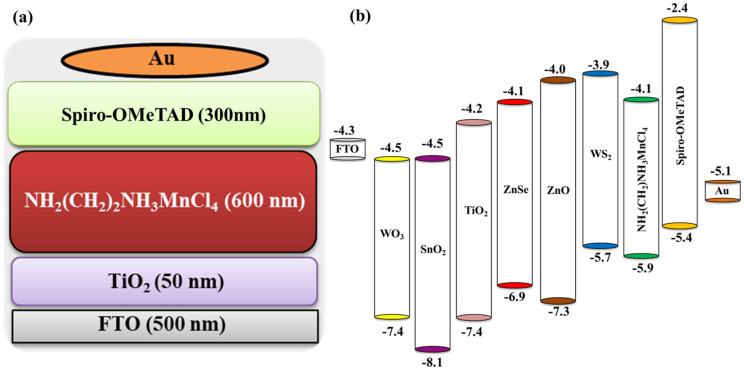
Pictorial representation showing device structure (**a**) and energy level; (**b**) diagram of PSCs.

We designed a mesoscopic device (FTO/TiO_2_/NH_3_(CH_2_)_2_NH_3_MnCl_4_/spiro-OMeTAD/Au) structure for the simulation of high-performance Pb-free PSCs (Scheme 1a). The energy level values of the light absorber layer, ETL, spiro-OMeTAD, and Au layers are displayed in Scheme 1b. Further, the Pb-free PSC device (FTO/TiO_2_/NH_3_(CH_2_)_2_NH_3_MnCl_4_/spiro-OMeTAD/Au) was developed under a controlled humidity (~30–40%). Fabrication details are provided in the supporting information. 

## 3. Results

### 3.1. Photovoltaic Performance of Simulated PSCs

**Figure 1:**
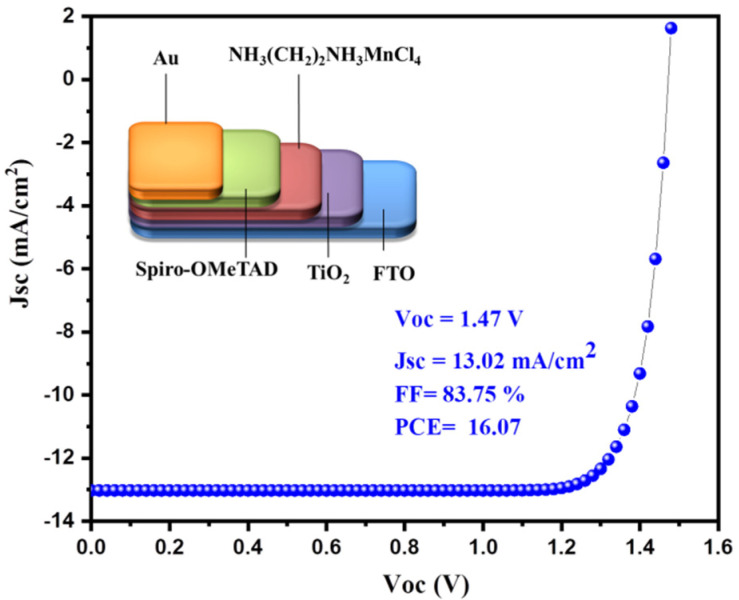
JV graph of FTO/TiO_2_ (50 nm)/NH_3_(CH_2_)NH_3_MnCl_4_ (250 nm)/Spiro-OMeTAD (300 nm)/Au.

The photovoltaic performance of the simulated PSCs devices were evaluated by J-V curves. Figure 1 shows the J-V graph of the numerically simulated PSC device (FTO/TiO_2_ (50 nm)/NH_3_(CH_2_)NH_3_MnCl_4_ (250 nm)/Spiro-OMeTAD (300 nm)/Au). The simulated device exhibited good Voc (1.47 V) and Jsc (13.02 mA/cm^2^). 

This enhanced Voc value indicated better electron transportation between the NH_3_(CH_2_)NH_3_MnCl_4_ and TiO_2_ layer, and an interesting PCE of 16.07% was obtained. This obtained PCE showed that NH_3_(CH_2_)NH_3_MnCl_4_ may be a potential Pb-free light absorber material for the development of PSCs. The thickness of the absorber (NH_3_(CH_2_)NH_3_MnCl_4_) layer plays an important role, which can significantly affect the photovoltaic activity of the developed PSCs. Therefore, we optimized the thickness of the NH_3_(CH_2_)_2_NH_3_MnCl_4_ to examine the effect of thickness on the photovoltaic performance of the simulated device. 

**Figure 2:**
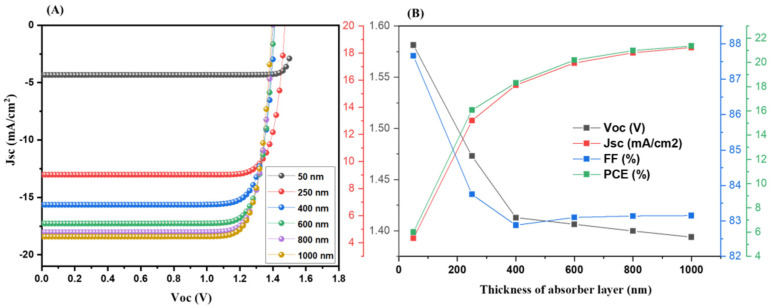
J-V curves (**A**) and photovoltaic parameters (**B**) of FTO/TiO_2_ (50 nm)/NH_3_(CH_2_)NH_3_MnCl_4_(varying)/Spiro-OMeTAD (300 nm)/Au.

We simulated the PSC device (FTO/TiO_2_ (50 nm)/NH_3_(CH_2_)NH_3_MnCl_4_(varying)/Spiro-OMeTAD (300 nm)/Au) by varying the thickness (50, 250, 400, 600, 800, and 1000 nm) of the NH_3_(CH_2_)_2_NH_3_MnCl_4_ layer. Figure 2A demonstrates the J-V graphs of the PSCs with various thicknesses of NH_3_(CH_2_)NH_3_MnCl_4_. The simulated results showed that the Voc and FF of the PSCs decrease, whereas the Jsc value increases with increasing thicknesses of NH_3_(CH_2_)_2_NH_3_MnCl_4_ from 50 nm to 1000 nm. However, the PCE is enhanced when the thickness of the NH_3_(CH_2_)_2_NH_3_MnCl_4_ layer increases (Figure 2B).

The high thickness of the NH_3_(CH_2_)_2_NH_3_MnCl_4_ layer had a more active absorber area, which resulted in the creation of more photons and increased Jsc value (Figure 2B). On the other side, a high thickness of the NH_3_(CH_2_)_2_NH_3_MnCl_4_ layer may allow higher carrier recombination, which may be responsible for decreased Voc value. Therefore, it is required to use the optimum thickness of the NH_3_(CH_2_)_2_NH_3_MnCl_4_ layer to overcome the issue of carrier recombination without compromising the PCE of the device. The observations showed that the PCE increases rapidly with the increasing thickness of the NH_3_(CH_2_)_2_NH_3_MnCl_4_ layer up to 600 nm thickness. Therefore, we used a 600 nm thickness of the NH_3_(CH_2_)_2_NH_3_MnCl_4_ layer for further simulation studies. The thickness of ETL and/or HTL plays an important role in PSCs. Hence, we optimized the thickness of TiO_2_ and spiro-OMeTAD layers. 

**Figure 3:**
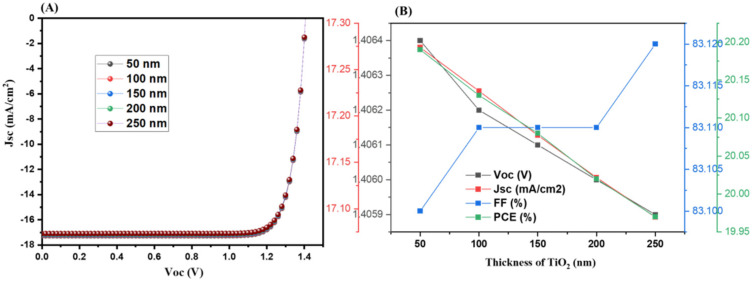
J-V curves (**A**) and photovoltaic parameters (**B**) of FTO/TiO_2_(varying)/NH_3_(CH_2_)NH_3_MnCl_4_ (600 nm)/Spiro-OMeTAD (300 nm)/Au.

**Figure 4:**
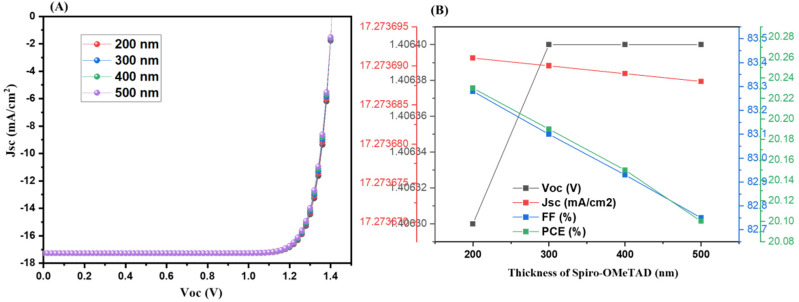
J-V curves (**A**) and photovoltaic parameters (**B**) of FTO/TiO_2_ (50 nm)/NH_3_(CH_2_)NH_3_MnCl_4_ (600 nm)/Spiro-OMeTAD(varying)/Au.

Figure 3A showed the J-V graphs of the FTO (500 nm)/TiO_2_(varying)/NH_3_(CH_2_)_2_NH_3_MnCl_4_ (600 nm)/spiro-OMeTAD (300 nm) devices. The obtained results indicated that the Voc, Jsc, and PCE of the PSCs devices decrease when the thickness of the TiO_2_ layer changes from 50 nm to 250 nm (Figure 3B). It is well-known that sheet resistance increases while conductivity decreases with the increasing thickness of ETL. Therefore, the optimum thickness of the TiO_2_ layer was found to be 50 nm for further investigations. The optimization of HTL is very important because of the direct contact between HTL and the counter electrode (Au). If the thickness of HTL is very thin, then the counter electrode may contact the light absorber layer. If the thickness of HTL is very thick, then it may increase sheet resistance and reduce the performance of the photovoltaic device. Thus, optimization of the thickness of HTL is of great importance to achieve the high efficiency of PSCs. Figure 4A showed the J-V curves of the FTO/TiO_2_ (50 nm)/NH_3_(CH_2_)NH_3_MnCl_4_ (600 nm)/Spiro-OMeTAD(varying)/Au with different thicknesses of spiro-OMeTAD. 

The simulation results (J-V graphs) showed that Voc increases whereas Jsc and PCE decrease when the thickness of the spiro-OMeTAD changes from 200 nm to 500 nm (Figure 4B). The increasing thickness of spiro-OMeTAD may increase sheet resistance which reduces the PCE of the PSCs. Thus, it is important to find out the optimum thickness of spiro-OMeTAD. The optimum thickness of 300 nm of spiro-OMeTAD was used for further photovoltaic investigations. The optimization efficiency with respect to the thickness of the absorber layer, ETL, and HTL are provided in Tables S3–S6 in the supporting material. 

**Figure 5:**
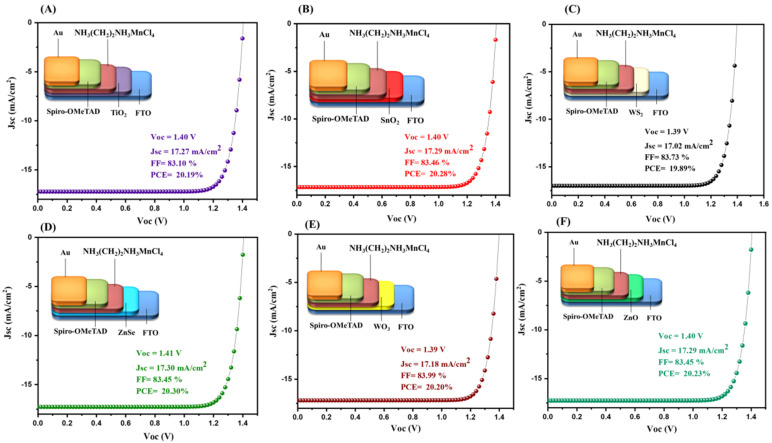
J-V curves (**A**–**F**) of FTO/ETL (50 nm)/NH_3_(CH_2_)NH_3_MnCl_4_ (600 nm)/Spiro-OMeTAD (300 nm)/Au.

The type of ETL has a great impact on the performance of the PSCs. Thus, the selection of suitable ETLs is necessary to develop high-performance PSCs. Therefore, we have employed tin oxide (SnO_2_), zinc oxide (ZnO), tungsten trioxide (WO_3_), tungsten disulfide (WS_2_), and zinc selenide (ZnSe) as the ETL for the simulation of high-performance PSCs. Figure 5B shows the J-V curve of the simulated PSCs with SnO_2_, such as ETL, which exhibited a PCE of 20.28%. This PCE was higher than that of the TiO_2_-based PSCs device (Figure 5A). In the case of WS_2_, PCE was decreased to 19.89% (Figure 5C), whereas ZnSe-based PSCs exhibited an improved PCE of 20.30% (Figure 5D). The photovoltaic performance of optimized PSCs with different ETLs have been summarized in Table S7. The WO_3_-based PSC device exhibited a PCE of 20.20% (Figure 5E), whereas ZnO-based PSCs showed a PCE of 20.23% (Figure 5F). The SnO_2_-based device exhibited a good PCE of 20.28%, but an improved PCE of 20.30% was obtained for ZnSe-based PSCs. Thus, it can be stated that ZnSe may be the most suitable and efficient ETL for the development of high-performance Pb-free PSCs. The highest PCE for ZnSe-based PSCs may be due to the high electron/hole mobility and better charge extraction/electron transportation. Moreover, the conduction band of ZnSe is in better alignment with the lowest unoccupied molecular orbital (LUMO) of NH_3_(CH_2_)NH_3_MnCl_4_. This well-matching of energy level values and high electron-hole mobility of ZnSe facilitates electron transfer and results in the enhanced photovoltaic performance of the PSCs. The external quantum efficiency (EQE) of the best-performing devices with different ETLs was also investigated. The EQE graphs of the PSCs with different ETLs have been presented in Figure S1. The obtained results showed that EQE covers the maximum visible region up to 700 nm.

**Figure 6:**
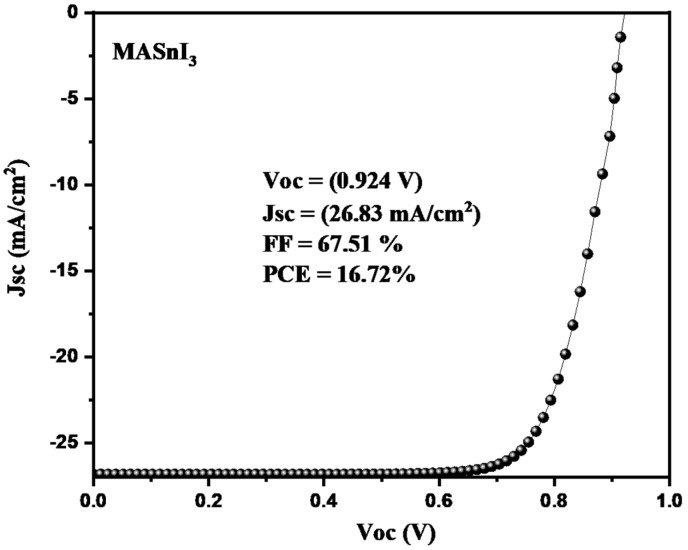
J-V graph of reference device (TiO_2_/MASnI_3_/spiro-OMeTAD) [29].

To verify our used calculations and software inputs, we also simulated PSCs (TiO_2_/MASnI_3_/spiro-OMeTAD) according to what was reported elsewhere [29]. The J-V graph of the simulated PSCs device has been presented in Figure 6. The obtained results showed the Voc of 0.92 f V with an FF of 67.51%. The simulated PSC (TiO_2_/MASnI_3_/spiro-OMeTAD) device also exhibits a Jsc and PCE of 26.83 mA/cm^2^ and 16.72%, respectively. Singh et al. [29] reported PSC devices with photovoltaic parameters of Jsc, FF (%), Voc, and PCE of 26.90 mA/cm^2^, 67.19, 0.924 V, and 16.71%, respectively. Our obtained results are almost identical to the previously reported PSC (TiO_2_/MASnI_3_/spiro-OMeTAD) device [29]. This suggested that our simulated results for NH_3_(CH_2_)NH_3_MnCl_4_-based PSCs are authentic. 

Recently, various research groups have adopted SCAP-1D for numerical simulation studies of PSCs. In this connection, Hima et al. [3] optimized the performance of CH_3_NH_3_GeI_3_-based PSCs and reported an interesting PCE of 13.30%. Raoui et al. [33] employed a double halide perovskite Cs_2_AgBiBr_6_ light absorber and reported a PCE of 5.15%, whereas 7.36% was achieved using FACsPb_0.5_Sn_0.5_I_3_. Sharma et al. [34] employed a Cs_2_AgBi_0.75_Sb_0.25_Br_6_ light absorber and reported a decent PCE of 10.01%. Ahmad et al. [35] reported a PCE of 15.06% using Cs_2_TiI_6_, whereas Ahmed et al. [36] obtained a PCE of 11.49% using a Cs_2_TiBr_6_ light absorber. In another work, Cs_2_TiBr_6_ was also employed as a light absorber, and the reported PCE was 8.51% [37]. Lee et al. [38] utilized CsSnBr_3_ and reported a PCE of 10.46% whereas 9.66% efficiency was reported for CsSnCl_3_-based PSCs [39]. In our case, we have achieved a higher PCE of 20.30%, which is comparable with recent works, as listed in Table 1. Therefore, it can be said that NH_3_(CH_2_)_2_NH_3_MnCl_4_ may be a potential absorber layer for the development of high-performance Pb-free PSCs. 

### 3.2. Photovoltaic Properties of Fabricated PSCs

**Figure 7:**
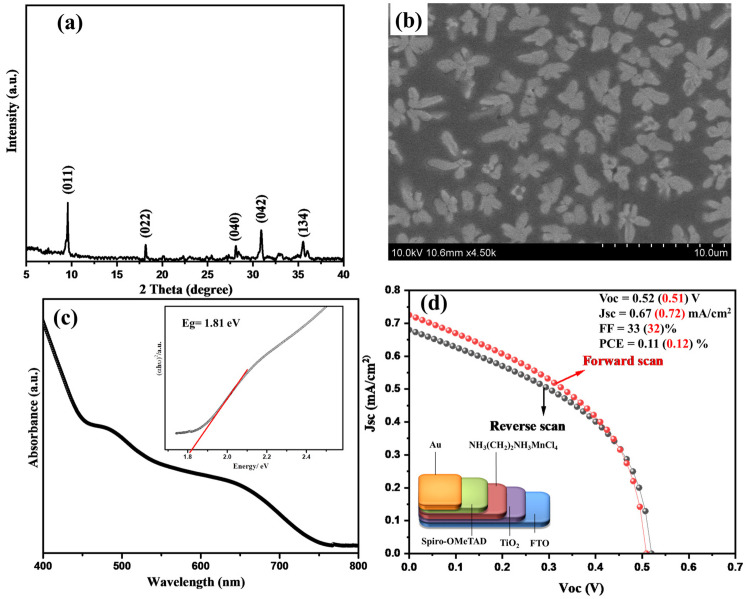
XRD (**a**), SEM (**b**), and UV-vis spectrum (**c**) of NH_3_(CH_2_)_2_NH_3_MnCl_4_ (Inset shows Tauc plot). J-V curve (**d**) of fabricated PSCs.

To further validate the simulated results, we have fabricated PSCs using NH_3_(CH_2_)_2_NH_3_MnCl_4_ as a light absorber layer, whereas TiO_2_ and spiro-OMeTAD were ETL and HTL, respectively. Firstly, we prepared a thin film of NH_3_(CH_2_)_2_NH_3_MnCl_4_ on FTO glass substrates by spin coating, and its physiochemical and optical properties were investigated. The recorded X-ray diffraction (XRD) pattern of the NH_3_(CH_2_)_2_NH_3_MnCl_4_ is presented in Figure 7a. The XRD pattern of NH_3_(CH_2_)_2_NH_3_MnCl_4_ showed a strong diffraction peak at ~9.6°, which indicated the presence of a crystalline nature. The obtained XRD pattern was well-matched with CCDC number 2015666. The surface morphology of the prepared NH_3_(CH_2_)_2_NH_3_MnCl_4_ film was also examined by employing scanning electron microscopy (SEM) analysis. The obtained SEM image of the NH_3_(CH_2_)_2_NH_3_MnCl_4_ film is presented in Figure 7b. The observations suggest that NH_3_(CH_2_)_2_NH_3_MnCl_4_ has a rough surface, which may be due to the rapid crystallization of NH_3_(CH_2_)_2_NH_3_MnCl_4_. The optical property of the NH_3_(CH_2_)_2_NH_3_MnCl_4_ was studied by using ultraviolet-visible (UV-vis)-absorption spectroscopy. The collected UV-vis spectrum of the NH_3_(CH_2_)_2_NH_3_MnCl_4_ is depicted in Figure 7c. The obtained results for NH_3_(CH_2_)_2_NH_3_MnCl_4_ exhibit an absorption band at the wavelength of 600–700 nm. The optical bandgap of the NH_3_(CH_2_)_2_NH_3_MnCl_4_ was determined by using the Tauc relation. The Tauc-plot of the NH_3_(CH_2_)_2_NH_3_MnCl_4_ is depicted in the inset of Figure 7c, which shows that NH_3_(CH_2_)_2_NH_3_MnCl_4_ has a band gap of ~1.81 eV. This obtained band gap is suitable for photovoltaic applications. Furthermore, we fabricated a PSC device (FTO/c-TiO_2_/m-TiO_2_/NH_3_(CH_2_)_2_NH_3_MnCl_4_/Spiro-OMeTAD/Au) under a controlled humidity (~30–40%). Further, the photovoltaic performance of the developed PSC device was evaluated by collecting a J-V graph under 1 sun conditions. The obtained J-V graph of the FTO/c-TiO_2_/m-TiO_2_/NH_3_(CH_2_)_2_NH_3_MnCl_4_/Spiro-OMeTAD/Au is presented in Figure 7d. The obtained results (J-V graph) showed a good Voc value of 510 mV but a lower Jsc of 0.71 mA/cm^2^ along with an FF value of 32%. Therefore, the obtained PCE was found to be 0.12%. This experimental PCE was poor compared to the theoretical findings, but we believe this poor performance of the fabricated device may be further enhanced by employing novel device architectures and controlling the rapid crystallization of NH_3_(CH_2_)_2_NH_3_MnCl_4_. The EQE of the fabricated PSC device was also collected. The obtained EQE of the NH_3_(CH_2_)_2_NH_3_MnCl_4_-based PSCs is depicted in Figure S2, which shows the light absorption between the wavelength of 300 and 600 nm. 

Previously, many research groups have developed Pb-free PSCs using different light absorbers. In 2015, Park et al. [40] developed PSCs using mixed halide (CH_3_NH_3_)_3_Bi_2_I_9_Cl_x_ perovskite-like material and reported a PCE of 0.003%. Cortecchia et al. [41] used a copper-based perovskite light absorber ((CH_3_NH_3_)_2_CuCl_2_Br_2_) and reported that the PCE was 0.017%. In another report, 1,6-hexanediammonium bismuth iodide was employed as the absorber material by Fabian et al. [42], and the obtained PCE was 0.027%. Ahmad et al. [23] reported a PCE of 0.1% using (CH_3_NH_3_)_3_Sb_2_I_9_, whereas 0.001% was obtained using [(CH_3_NH_3_)_3_Bi_2_Cl_9_]*_n_* as a light absorber [26]. Thind et al. [43] utilized a KBaTeBiO_6_ light absorber and fabricated a PSC device that showed a PCE of 0.06%. Mixed halide (NH_4_)_3_Sb_2_I_3_Br_6_ was used by Zuo et al. [44], and the developed device exhibited a poor PCE of 0.06%. In some other reports, bismuth-based absorber layers have also been used for the construction of Pb-free PSCs. In this report, we demonstrated the role of NH_3_(CH_2_)_2_NH_3_MnCl_4_ as an absorber layer, which exhibited decent photovoltaic performance, as listed in Table 2. It can be clearly seen from Table 2 that the PCE of NH_3_(CH_2_)_2_NH_3_MnCl-based fabricated PSC device is poor (0.12%) but showed a decent Voc. The absorber layer should have a low band gap for better light absorption. Although NH_3_(CH_2_)_2_NH_3_MnCl_4_ has a good optical band gap, we believe that the poor PCE and Jsc of the NH_3_(CH_2_)_2_NH_3_MnCl_4_-based fabricated PSC device may be attributed to the poor morphological characteristics and fast crystallization process. The rapid crystallization of perovskite films has been a major concern in developing high-performance photovoltaic devices. The preparation of pin-hole-free and high-quality thin films with large grain boundaries is of great importance for the construction of highly efficient PSC devices. Previously, two-step deposition methods or anti-solvents such as chlorobenzene have been used for the preparation of high-quality thin films of perovskite materials. Some new preparation methods need to be developed to exert tight control on the crystallization process. It is also important to study the mechanism of thin film formation, charge-separation/recombination at interfaces/grain boundaries to further improve the performance of NH_3_(CH_2_)_2_NH_3_MnCl_4_-based PSCs. We believe that, if systematic investigations are implemented, significant improvements in the performance of the NH_3_(CH_2_)_2_NH_3_MnCl_4_-based PSCs can be seen in the near future. 

## 4. Conclusions

Finally, it can be summarized that highly efficient Pb-free PSCs have been simulated via SCAPS-1D. Further, we optimized the thickness of electron, absorber, and hole transport material layers. The numerically simulated PSCs exhibited good efficiency of 20.30% via SCAPS-1D. Various electron transport layers (WS_2_, ZnO, SnO_2_, TiO_2_, ZnSe, and WO_3_) were employed, and ZnSe-based PSCs showed the highest efficiency, which may be due to the high electron-hole mobility and suitable energy level values of ZnSe. Furthermore, NH_3_(CH_2_)_2_NH_3_MnCl_4_ was prepared, and its optical band gap was found to be 1.81 eV, which is suitable for a light absorber layer. Further, PSCs were fabricated, and the developed device exhibited a good Voc of 510 mV with a poor PCE of 0.12%. We believe this PCE may be further enhanced in the future by using/introducing some new device architectures or fabrication methods.

## Figures and Tables

**Table 1:** Comparison of performance of simulated NH_3_(CH_2_)_2_NH_3_MnCl_4_-based PSCs with recently published simulated work [3,33,34,36,37,38,39].

Absorber	Voc (V)	FF (%)	Jsc (mA/cm^2^)	PCE (%)	References
**NH_3_(CH_2_)_2_NH_3_MnCl_4_**	**1.77**	**83.45**	**17.30**	**20.30**	**This study**
Cs_2_TiBr_6_	1.12	73.59	10.25	8.51	[37]
Cs_2_TiBr_6_	1.53	86.45	8.66	11.49	[36]
Cs_2_AgBiBr_6_	1.14	58.70	14.9	10.01	[34]
Cs_2_AgBi_0.75_Sb_0.25_Br_6_	0.97	47.43	11.16	5.15	[33]
CsSnCl_3_	0.87	56	19.82	9.66	[39]
CsSnBr_3_	0.85	58	21.23	10.46	[38]
CH_3_NH_3_GeI_3_	0.93	60.75	23.44	13.30	[3]

**Table 2:** Comparison of Voc, Jsc, FF, and PCE of the fabricated NH_3_(CH_2_)_2_NH_3_MnCl_4_-based PSCs with recently published experimental works [23,26,40,41,42,43,44,45,46,47,48,49].

Absorber	Voc (V)	FF (%)	Jsc (mA/cm^2^)	PCE (%)	References
**NH_3_(CH_2_)_2_NH_3_MnCl_4_**	**0.51**	**32**	**0.72**	**0.12**	**This study**
(CH_3_NH_3_)_3_Sb_2_I_9_	0.69	40	0.39	0.1	23
[(CH_3_NH_3_)_3_Bi_2_Cl_9_]_n_	0.430	-	-	0.001	26
(CH_3_NH_3_)_3_Bi_2_I_9_Cl_x_	0.04	38	0.18	0.003	40
(CH_3_NH_3_)_2_CuCl_2_Br_2_	0.29	28	0.216	0.017	41
1,6-hexanediammonium bismuth iodide	0.384	-	0.124	0.027	42
KBaTeBiO_6_	0.54	58	0.09	0.06	43
(NH_4_)_3_Sb_2_I_3_Br_6_	0.67	44	0.20	0.06	44
Cs_3_Bi_2_I_9_	0.74	51	3.42	1.26	45
Cs(Bi_0.7_Sb_0.3_)_3_I_10_	0.81	34.8	5.47	1.54	46
AgBi_2_I_7_	0.62	70	4.83	2.12	47
BiI_3_	0.587	38	4.54	1.013	48
BiI_3_	0.31	40	0.34	0.49	49

## Data Availability

Not applicable.

## Supplementary Materials

The following supporting information can be downloaded at: https://www.mdpi.com/article/10.3390/nano12193407/s1, Figure S1: QE curves (a–f) of FTO/ETL(50 nm)/NH_3_(CH_2_)NH_3_MnCl_4_(600 nm)/Spiro-OMeTAD(300 nm)/A; Figure S2: EQE curve of the fabricated PSCs deviceTable S1. Numerical parameters of different materials for device simulation; Table S2: Numerical parameters of different ETLs for device simulation; Table S3: Effect of thickness of light absorber layer; Table S4: Effect of thickness of TiO_2_; Table S5: Effect of thickness of Spiro-OMeTAD; Table S6: Effect of different ETL layers.
