# Supplementary material for: Numerical Simulation of NH3(CH2)2NH3MnCl4 Based Pb-Free Perovskite Solar Cells Via SCAPS-1D
Source: Nanomaterials (Basel). 2022 Sep 28;12(19):3407. doi: 10.3390/nano12193407 (PMC9565589; doi:10.3390/nano12193407)
Supplement: Supplementary file 1 [file nanomaterials-12-03407-s001.zip › nanomaterials-1927726-supplementary.pdf]

Supporting Information

Numerical simulation of $NH_3(CH_2)_2NH_3MnCl_4$ based Pb-free perovskite solar cells via SCAPS-1D

Khursheed Ahmad[a], Waseem Raza[b], Rais Ahmad Khan,[c], Ali Alsalme,[c,*] and Haekyoung Kim[a,*]

[a]*School of Materials Science and Engineering, Yeungnam University, Gyeongsan, Republic of Korea, 38541*
[b]*Department of Chemical Engineering, Indian Institute of Technology Delhi, Hauz Khas, New Delhi, 110016, India*
[c]*Department of Chemistry, College of Science, King Saud University, Riyadh-11451, Kingdom of Saudi Arabia*
* Correspondence: author email: H. Kim (hkkim@ynu.ac.kr), A. Alsalme (aalsalme@ksu.edu.sa)

1. Experimental Section

1.1. Chemicals

Fluorine doped tin oxide (FTO) substrates, hydrochloride (HCl, 37%) ethylene diamine dihydrochloride ($NH_2(CH_2)_2NH_2·2HCl$), 2,2′,7,7′-tetrakis-(*N,N*-di-4-methoxyphenylamino)-9,9′-spirobifuorene (spio-OMeTAD), lithium bis(trifluoromethylsyfonyl)imide salt (L-TFSI), and tert-butylpyridine (t-BP) were purchased from Sigma Aldrich. Chlorobenzene, and manganese dichloride ($MnCl_2$), were purchased from Merck and Fisher scientific. Mesoporous titanium dioxide (18 NRT, TiO_2 paste) was purchased from Dyesol. All the other chemicals and solvents were purchased from Sigma and Alfa Aesar.

1.2. Instrumental

X-ray diffraction pattern (XRD) of $NH_3(CH_2)_2NH_3MnCl_4$ was recorded on X′pert Pro X-ray diffractometer. Varian Ultraviolet-visible (UV-vis) spectrophotometer, model: Carry100 was used to record the UV-vis absorption spectrum of $NH_3(CH_2)_2NH_3MnCl_4$. Scanning electron microscope (SEM) model no. S-4800 was used to capture the SEM image of $NH_3(CH_2)_2NH_3MnCl_4$. Photocurrent-voltage (J-V) graphs of the fabricated PSCs device was obtained under 1 sun conditions (AM 1.5 G, and 100 mW/cm^2 illuminations) on solar simulator (Photo Emission Tech).

1.3. Fabrication of PSCs device

In first step, FTO substrates were patterned using zinc powder and 2 molar HCl which were further cleaned with mild detergent liquid, acetone, 2-propanol, and deionized water using ultrasonication for 15 min each. The TiO_2 compact layer (c-TiO_2) was prepared on FTO using titanium diisopropoxide bis(acetylacetatonate) as precursor whereas mesoporous-TiO_2 (m-TiO_2) layer was deposited using 18 NRT TiO_2 paste. The c-TiO_2 was deposited on FTO with spin coating of titanium diisopropoxide bis(acetylacetatonate) dissolved in ethanol (spin speed=4000 rpm; time=30 sec) and annealed at 450 ºC for 30 min. Further, m-TiO_2 layers was deposited on annealed FTO/c-TiO_2 (spin speed= 4000 rpm; time=30 sec), and annealed at 450 ºC for 30 min. Further, 20 wt% of $NH_2(CH_2)_2NH_2.2HCl$ and $MnCl_2$ (ratio=1:1) were dissolved in 2 mL dimethyl sulfoxide (DMSO) and stirred at 70 ºC overnight. The above stirred precursor solution was spin coated on the annealed FTO/c-TiO_2/m-TiO_2 (spin speed=4000 rpm; time=30 sec), and heated at 70 ºC for 30 min. Hole transport layer (HTL) was deposited on to the fabricated electrode (FTO/c-TiO_2/m-TiO_2/$NH_3(CH_2)_2NH_3MnCl_4$) (spin speed=3000 rpm; time=30 sec). The HTL was prepared by dissolving spiro-OMeTAD in chlorobenzene (30 mg/mL) with additives (Li-TFSI and t-BP). Finally, gold (Au) metal contact was deposited using thermal evaporation method. The fabricated PSCs device has been labelled as FTO/c-TiO_2/m-TiO_2/$NH_3(CH_2)_2NH_3MnCl_4$/spiro-OMeTAD/Au.

Table S1. Numerical parameters of different materials for device simulation [21, 27, 29-32].

Parameters	FTO	TiO_2	$NH_3(CH_2)_2NH_3MnCl_4$	spiro-OMeTAD
Thickness (nm)	500	varying	varying	varying
Band Gap (eV)	3.5	3.2	1.81	3

Electron affinity (eV)	4	4.2	4.17	2.45
Dielectric permittivity	9	10	6	3
CB effective density of states (1 cm^{-3})	2.2 x 10^{18}	2.2 x 10^{18}	2 x 10^{18}	2.2 x 10^{18}
VB effective density of states (1 cm^{-3})	1.8 x 10^{19}	1.8 x 10^{19}	1 x 10^{19}	1.8 x 10^{19}
Electron thermal velocity (cm S^{-1})	1 x 10^7	1 x 10^7	1 x 10^6	1 x 10^7
Hole thermal velocity (cm S^{-1})	1 x 10^7	1 x 10^7	1 x 10^6	1 x 10^7
Electron mobility (cm^2 VS^{-1})	20	100	1.6 x 100	2 x 10^{-4}
Hole mobility (cm^2 VS^{-1})	10	25	1.6 x 100	2 x 10^{-4}
Shallow uniform donor density ND (1 cm^{-3})	2 x 10^{19}	1 x 10^{19}	-	-
Shallow uniform acceptor density NA (1 cm^{-3})	-	-	3.2 x 10^{15}	2 x 10^{18}
Defect type	Neutral	Neutral	Neutral	Neutral
Deep defect density (Nt)	1 x 10^{15}	1 x 10^{15}	1 x 10^{15}	1 x 10^{15}

Table S2. Numerical parameters of different ETLs for device simulation [21,27,29-32].

**Parameters**	**WS_2**	**ZnO**	**SnO_2**	**ZnSe**	**WO_3**
Thickness (nm)	50	50	50	varying	50
Band Gap (eV)	1.8	3.3	3.5	2.81	2.92
Electron affinity (eV)	3.95	4	4.4	4.09	4.59
Dielectric permittivity	13.60	9	9	8.6	5.76
CB effective density of states (1 cm^{-3})	1 x 10^{18}	3.7 x 10^{18}	2.2 x 10^{18}	2.2 x 10^{18}	1.96 x 10^{19}
VB effective density of states (1 cm^{-3})	1 x 10^{18}	1.8 x 10^{19}	1.8 x 10^{19}	1.8 x 10^{19}	1.96 x 10^{19}
Electron thermal velocity (cm S^{-1})	1 x 10^7	1 x 10^7	1 x 10^7	1 x 10^7	1 x 10^7
Hole thermal velocity (cm S^{-1})	1 x 10^7	1 x 10^7	1 x 10^7	1 x 10^7	1 x 10^7
Electron mobility (cm^2 VS^{-1})	50	100	20	110	10
Hole mobility (cm^2 VS^{-1})	50	25	10	400	10
Shallow uniform donor density ND (1 cm^{-3})	1 x 10^{18}	5 x 10^{17}	2 x 10^{19}	1 x 10^{18}	3.68 x 10^{19}
Shallow uniform acceptor density NA (1 cm^{-3})	-	-	-	-	-
Deep defect density (Nt)	1 x 10^{15}	1 x 10^{15}	1 x 10^{15}	1 x 10^{15}	1 x 10^{15}
Defect type	Neutral	Neutral	Neutral	Neutral	Neutral

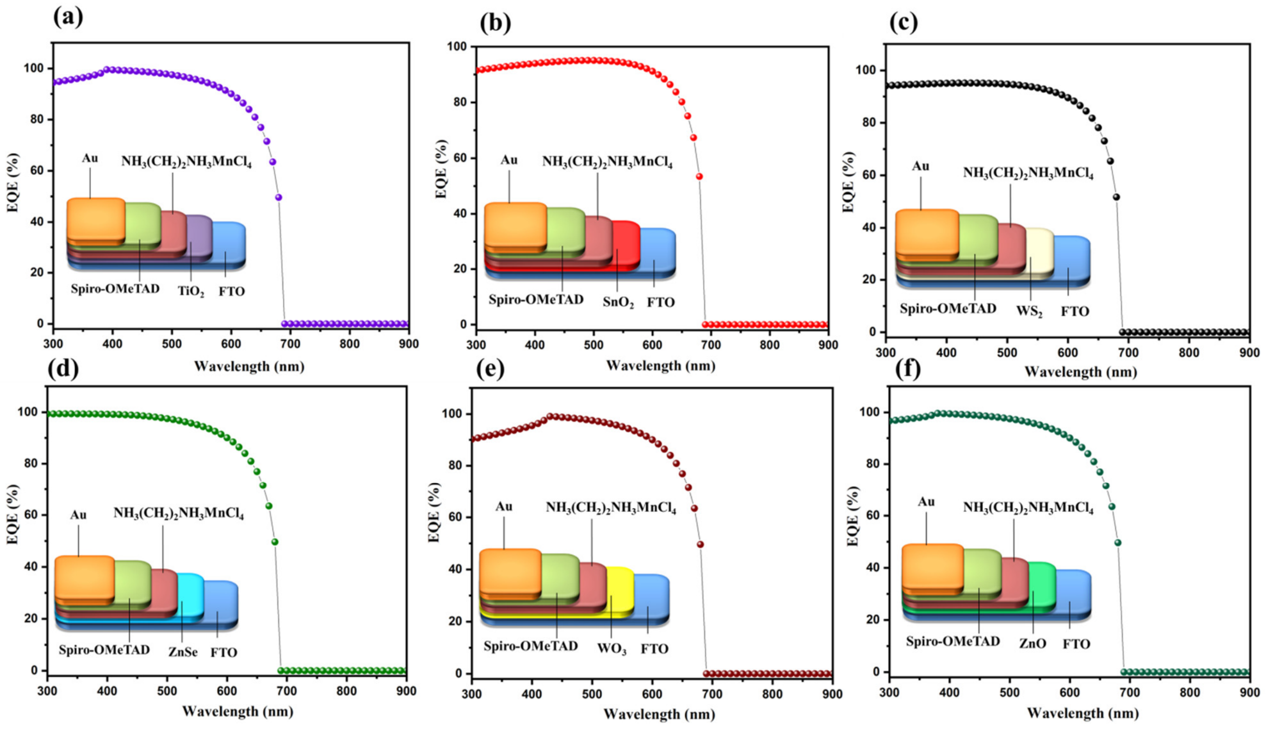

Figure S1. QE curves (a-f) of FTO/ETL(50nm)/$NH_3(CH_2)NH_3MnCl_4$(600nm)/Spiro-OMeTAD(300nm)/Au.

Table S3. Effect of thickness of light absorber layer.

Thickness of $NH_2(CH_2)_2NH_3MnCl_4$ (nm)	Spiro-OMeTAD (nm)	Thickness of TiO_2 (nm)	Thickness of FTO (nm)	Voc (V)	Jsc (mA/cm^2)	FF (%)	PCE (%)
50	300	50	500	1.5814	4.331556	87.66	6.0
250	300	50	500	1.4730	13.027064	83.75	16.07
400	300	50	500	1.4129	15.642162	82.88	18.32
600	300	50	500	1.4064	17.273690	83.10	20.19
800	300	50	500	1.3998	18.028891	83.14	20.98
1000	300	50	500	1.3939	18.416579	83.15	21.35

Table S4. Effect of thickness of TiO_2.

Thickness of $NH_2(CH_2)_2$ NH_3MnCl_4 (nm)	Spiro-OMeTAD (nm)	Thickness of TiO_2 (nm)	Thickness of FTO (nm)	Voc (V)	Jsc (mA/cm^2)	FF (%)	PCE (%)
600	300	50	500	1.4064	17.273690	83.10	20.19
600	300	100	500	1.4062	17.226895	83.11	20.13
600	300	150	500	1.4061	17.179245	83.11	20.08
600	300	200	500	1.4060	17.133710	83.11	20.02
600	300	250	500	1.4059	17.091084	83.12	19.97

Table S5. Effect of thickness of Spiro-OMeTAD.

Thickness of $NH_2(CH_2)_2$ NH_3MnCl_4 (nm)	Spiro-OMeTAD (nm)	Thickness of TiO_2 (nm)	Thickness of FTO (nm)	Voc (V)	Jsc (mA/cm^2)	FF (%)	PCE (%)
600	200	50	500	1.4063	17.273691	83.28	20.23
600	300	50	500	1.4064	17.273690	83.10	20.19
600	400	50	500	1.4064	17.273689	82.93	20.15

600	500	50	500	1.4064	17.273688	82.75	20.10

Table S6. Effect of different ETL layers.

Thickness of $NH_2(CH_2)_2 NH_3MnCl_4$ (nm)	Spiro-OMeTAD (nm)	Type of ETL (50 nm)	Thickness of FTO (nm)	Voc (V)	Jsc (mA/cm^2)	FF (%)	PCE (%)
600	300	TiO_2	500	1.4064	17.273690	83.10	20.19
600	300	ZnSe	500	1.4064	17.296821	83.45	20.30
600	300	WS_2	500	1.3970	17.002287	83.73	19.89
600	300	ZnO	500	1.4064	17.293874	83.45	20.23
600	300	SnO_2	500	1.4050	17.291855	83.46	20.28
600	300	WO_3	500	1.3990	17.189589	83.99	20.20

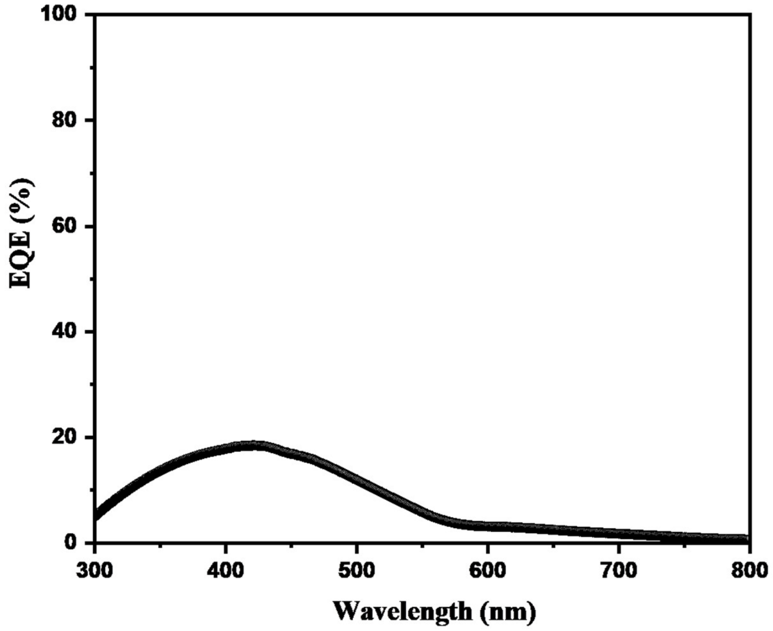

Figure S2. EQE curve of the fabricated PSCs device.
